# Meibomian Gland Dysfunction Determines the Severity of the Dry Eye Conditions in Visual Display Terminal Workers

**DOI:** 10.1371/journal.pone.0105575

**Published:** 2014-08-21

**Authors:** Huping Wu, Yuqian Wang, Nuo Dong, Fan Yang, Zhirong Lin, Xumin Shang, Cheng Li

**Affiliations:** Xiamen Eye Center of Xiamen University, Xiamen, Fujian, China; Tokai University, Japan

## Abstract

**Objective:**

To explore meibomian gland dysfunction (MGD) may determine the severity of dry eye conditions in visual display terminal (VDT) workers.

**Methodology:**

Prospective, case-control study carried out in China.106 eyes of 53 patients (VDT work time >4 hour per day) were recruited as the Long time VDT group; 80 eyes of 40 control subjects (VDT work time ≤4 hour per day) served as the Short time VDT group. A questionnaire of Ocular Surface Disease Index (OSDI) and multiple tests were performed. Three dry eye tests: tear film breakup time (BUT), corneal fluorescein staining, Schirmer I test; and three MGD parameters: lid margin abnormality score, meibum expression assessment (meibum score), and meibomian gland dropout degree (meiboscore) using Keratograph 5 M.

**Principal Findings:**

OSDI and corneal fluorescein score were significantly higher while BUT was dramatically shorter in the long time VDT group than the short time VDT group. However, the average of Schirmer tear volumes was in normal ranges in both groups. Interestingly, the three MGD parameters were significantly higher in the long time VDT group than the short time one (P<0.0001). When 52 eyes with Schirmer <10 mm and 54 eyes with Schirmer ≥10 mm were separated from the long time VDT workers, no significant differences were found between the two subgroups in OSDI, fluorescein staining and BUT, as well as the three MGD parameters. All three MGD parameters were positively correlated with VDT working time (P<0.0001) and fluorescein scores (P<0.0001), inversely correlated with BUT (P<0.05), but not correlated with Schirmer tear volumes in the VDT workers.

**Conclusions:**

Our findings suggest that a malfunction of meibomian glands is associated with dry eye patients in long term VDT workers with higher OSDI scores whereas some of those patients presenting a normal tear volume.

## Introduction

In the global information age with overwhelming development of digital devices, working related to visual display terminals (VDT) is increasing dramatically, which is also accompanied by a higher incidence of health problems, such as ocular discomfort, musculoskeletal disorders, and mental health [1]. The adverse effect of VDT working that causes ocular symptoms is an important public health issue that may interfere with quality of life, work performance and productivity [2]. Long term of VDT work, especially for more than 4 hours daily, was observed to be associated with a high incidence of dry eye disease [3], [4]. The development of ocular complaints among VDT workers was also reported to be correlated with the dysfunction of meibomian glands (MGD) [5], which is considered as one of the major causes of ocular complaints, inflammation and other ocular surface disorders [6].

The meibomian glands in the eyelids, which synthesis and secrete lipids for the superficial tear film layer, play an important role in maintaining a healthy ocular surface through reducing tear evaporation [7]. Dysfunction of meibomian glands, characterized by obstruction of gland orifices, changes of glandular secretion (meibum) and lid margins, leads to alternation of lipid layer and tear film instability, which may cause a variety of ocular surface disorders, in particularly, dry eye [8]. MGD has been generally considered a main reason for evaporative dry eye, and may be correlated with aqueous-deficient dry eye [9]. The use of VDT that make us keep eyes open widely has been recognized to decrease the frequency of blinking, and accelerate tear evaporation [10]. Very recently, Uchino and colleagues [11] observed that VDT-related dry eye had short tear film breakup time and increased corneal fluorescent staining but normal values of Schirmer test. However, it is not clear how important, and what mechanism is the meibomian gland function in dry eye conditions, especially in VDT workers who secrete normal values of aqueous tear. There was lack of published data and figures about morphological changes of meibomian glands in these subjects. Therefore we hypothesized that VDT working results in eye discomfort through its effects on meibomian glands, and that MGD may play a more significant role than aqueous tear volumes in determining the severity of dry eye conditions.

We have recently developed a quick and patient-friendly examination to visualize and photograph images of meibomian gland structure by OCULUS Keratograph 5 M technology. Our study evaluated the morphological characteristics and function of meibomian glands using this non-contact imaging device in a relatively large population of VDT workers. This clinical investigation for the first time revealed an important phenomenon that meibomian gland dysfunction not only associates but also determines the severity of the dry eye conditions in long term workers at visual display terminal.

## Patients and Methods

### Patients

The patient group for study was consisted of dry eye suffers working at a VDT for more than one year with mild to severe discomfort symptoms evaluated by a questionnaire of Ocular Surface Disease Index (OSDI), the OSDI score of each subject was ≥13 [12]. These subjects were divided into two groups based on their working time on VDT: the long time VDT group, 106 eyes of 53 consecutive patients who work at a VDT for more than 4 hours per day for 2–17 years (22 males and 31 females, age ranged from 20 to 47 years, mean ± standard deviation 31.79±5.53); and the short time VDT group serving as controls, 80 eyes of 40 age- and gender-matched patients working at a VDT for less than or equal to 4 hours per day for 1–10 years (22 males and 18 females; age ranged in 23–52 years, 30.58±5.83). All patients were examined at the affiliated Xiamen Eye Center of Xiamen University (Xiamen, Fujian, China), between January and October 2013. Before examination, each patient signed the written informed consent. All investigations adhered to the tenets of the Declaration of Helsinki and were approved by the Ethics Committee of Xiamen Eye Center. The patients who wear contact lens, uninterrupted use any kind of eye drops, have a history of allergic or infectious conjunctivitis, blepharitis, ocular surgery or trauma, and any systemic diseases or treatments that may affect the quality and stability of tear film were excluded from this study.

### Clinic examination

Each patient completed a questionnaire of OSDI for assessment of ocular surface symptoms and the severity of dry eye. Then the following examinations were carried out sequentially: examinations of lid margins by slit-lamp; measurements of tear film break-up time (BUT) and corneal fluorescein staining after fluorescein instillation; 30 minutes later, tear production was examined by Schirmer I test (measured without topical anesthetic); expression of meibum; and grading of meibomian gland loss by Keratograph 5 M. All examinations were completed on a separated day, and evaluated in the same darkened room by the same ophthalmologist.

### Evaluation of dry eye conditions

As we described previously [13], the tear film BUT was calculated for a mean value after 3 successive measurements, the value of Schirmer I test was depended on the moist length of the strip. Corneal fluorescein staining was graded from 0 to 12, a sum of the scores of corneal four quadrants, which were scored individually as 0 (no staining), 1 (mild staining with a few scattered dots of stains), 2 (moderate staining between 1 and 3), and 3 (severe staining with confluent stains or corneal filaments) [14].

### Evaluation of meibomian gland dysfunction

The following three parameters were the most commonly used methods to evaluate the morphological characteristics and function of meibomian glands in clinical practice: abnormalities of lid margins, expression of meibum, and gland dropout degree visualized by meibography [8]. Lid margin abnormality was recorded according to the existence of the following four signs [15]: lid margin irregular, vascular engorgement, glandular orifices obstruction, and anterior or posterior displacement of the mucocutaneous junction, the scored was from 0 to 4.

For assessing the expression of meibum semiquantitatively, the center of upper tarsus was expressed by thumb, the meibum score was graded as follows [16]: 0, clear meibum expressed easily; 1, cloudy meibum expressed gently; 2, cloudy meibum can be expressed with more than moderate pressure; and 3, no meibum can be expressed even with hard pressure.

Meibomian gland morphology was observed by Keratograph 5 M (OCULUS, Wetzlar, Germany), a noncontact, placido ring-based corneal topographer [17], [18]. The images of meibomian gland structure can be captured after eyelids eversion. This following meibomian gland dropout degree was graded for each eyelid as meiboscore [15]: grade 0 (no loss of meibomian glands), grade 1 (loss <33% of the whole glands area), grade 2 (loss area between 33% and 67%), and grade 3 (loss >67% of the whole area). The meiboscore of each eye was calculated as the sum of the scores from both upper and lower eyelids, which made the total meiboscore per eye to a range of 0–6. In this study, meiboscore per eye was presented in each group for comparison.

### Statistical Analysis

All statistical analyses were performed by SPSS V.13.0 software (SPSS Inc.; Chicago, Illinois, USA). Sex ratio among the two groups was evaluated using Chi-square test. Mann–Whitney U test was applied to compare the other parameters among the two groups and subgroups. When estimating the correlations between various factors, Spearman correlation analysis was used. The differences were regarded statistically significant when the value of *P*<0.05.

## Results

### VDT workers suffer dry eye disease with normal tear volumes

The general information and tear parameters in these two groups of VDT workers are shown in Table 1. The mean age and sex ratio between long and short time VDT groups showed no significant differences. The average time of working on the screen was 8.31±2.31 (range, 5–14) hours daily for 7.91±3.81 (2–17) years in the long time VDT group, much higher than 3.20±0.88 (1–4) hours daily for 5.25±2.85 (1–10) years in the short time VDT group. In the long time VDT group, OSDI score and corneal fluorescein staining presented significantly higher (*P*<0.01) while the tear film BUT showed dramatically shorter (*P*<0.001) compared with the short time VDT group. Interestingly, the average of tear volumes as measured by Schirmer test were in the normal ranges in both long (11.53±8.32 mm) and short time (12.88±9.66 mm) VDT groups.

**Table 1:** Patient Information and Tear Parameters in the Long and Short Time VDT Workers.

Parameters	Short time VDT (n = 80)	Long time VDT (n = 106)	*P*
Age (yr)	30.58±5.83 (23–52)	31.79±5.53 (20–47)	0.053
Sex ratio (male/female)	22/18	22/31	0.197
VDT working time (h/day)	3.20±0.88 (1–4)	8.31±2.31 (5–14)	<0.0001
VDT working years	5.25±2.85 (1–10)	7.91±3.81 (2–17)	<0.001
OSDI	29.23±10.98	37.38±18.23	<0.01
BUT (s)	6.71±2.66	4.92±2.41	<0.001
fluorescein score	1.46±1.67	3.73±2.98	<0.0001
Schirmer (mm)	12.88±9.66	11.53±8.32	0.554

Note: Data presented as Mean ± SD (ranges) of Values; n = number of eye; No statistic difference in tear production by Schirmer test between 2 groups (*P* = 0.554).

### Meibomian gland function in the long and short time VDT groups with normal tear volumes

**Figure 1:**
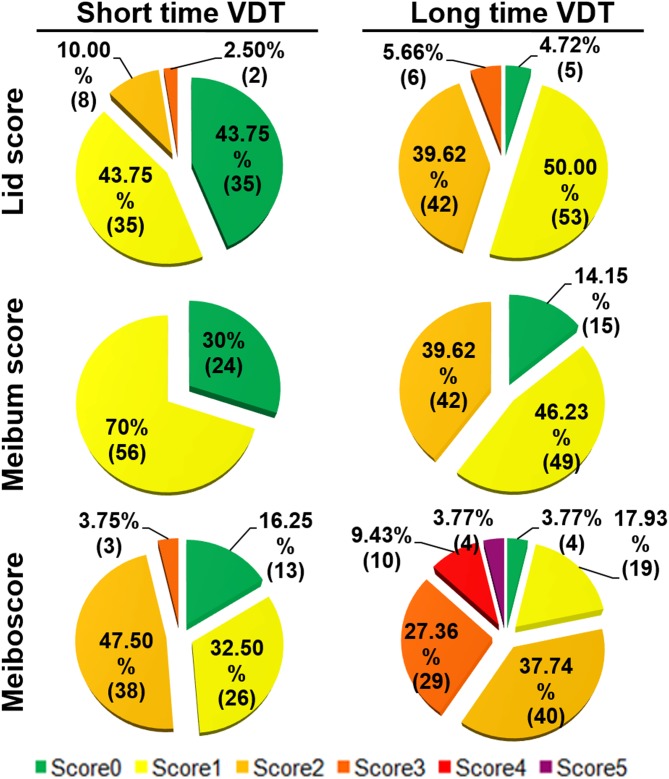
The Detailed Distributions of Each Meibomian Gland Parameter in the two groups of VDT Workers. Data presented as percentage (case number).

To investigate the potential mechanism why the workers of long time VDT group who secrete normal range of tear volumes suffer more severe dry eye symptoms than short time VDT group, the meibomian gland function was examined. Table 2 shows the incidence of three meibomian gland parameters in the long and short time VDT groups. Lid margin abnormality (score ≥2) was seen in 48 eyes (45.28%) in the long time VDT group while only 10 eyes (12.5%) in the short time VDT group. Cloudy meibum was observed in 91 eyes (85.85%) and 56 eyes (70%) in these two groups respectively. Apparent meibomian gland dropout (meiboscore ≥3) was seen in 43 eyes (40.56%) and 3 eyes (3.75%) respectively in the long and short time VDT groups. The detailed distributions of each parameter in these 2 groups were shown in Figure 1.

**Table 2:** Incidences of Meibomian Gland Parameters in the Long and Short Time VDT Workers.

	Short time VDT (n = 80)	Long Time VDT (n = 106)
**Lid margin score**		
0	35 (43.75%)	5 (4.72%)
1	35 (43.75%)	53 (50%)
2	8 (10%)	42 (39.62%)
3	2 (2.50%)	6 (5.66%)
4	0 (0%)	0 (0%)
**Meibum score**		
0	24 (30%)	15 (14.15%)
1	56 (70%)	49 (46.23%)
2	0 (0%)	42 (39.62%)
3	0 (0%)	0 (0%)
**Meiboscore**		
0	13 (16.25%)	4 (3.77%)
1	26 (32.50%)	19 (17.93%)
2	38 (47.50%)	40 (37.74%)
3	3 (3.75%)	29 (27.36%)
4	0 (0%)	10 (9.43%)
5	0 (0%)	4 (3.77%)
6	0 (0%)	0 (0%)

Note: Data presented as case number (percentage).

**Figure 2:**
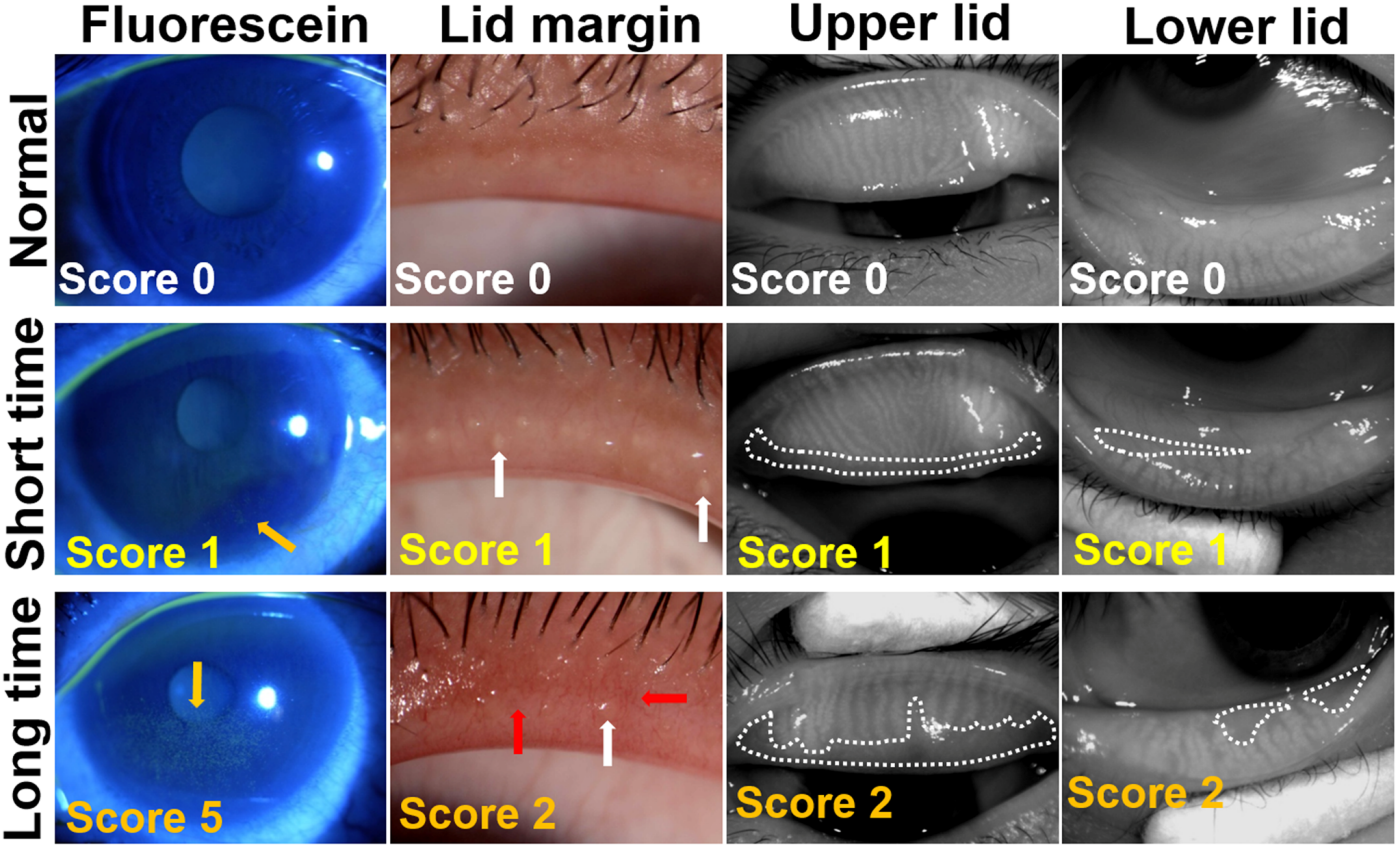
Representative images of fluorescein staining (Fluorescein), lid margin and meibography of upper lid and lower lid in patients from long or short time VDT groups. The score of each image is noted at bottom left. The upper panel shows normal eyes with score 0; the middle panel shows eyes from short time VDT group: the right eye from a 23-year-old female, VDT working time: 3 h/d; OSDI: 31.25; BUT: 10 s; Schirmer test: 15 mm; meibum score were 1. The bottom panel shown eyes from long time VDT group: the left eye from a 22-year-old female, VDT working time: 8 h/d; OSDI: 60; BUT: 4 s; Schirmer test: 15 mm; the meibum score was 2. Corneal fluorescein staining is noted by yellow arrows. Vascular engorgement is outlined by red arrow and plugged meibomian gland orifice is outlined by white arrow. The areas of meibomian gland dropout are encircled with dotted white lines.

The mean values and standard deviations of three parameters of meibomian gland were further analyzed and compared between these two groups. As summarized in Table 3, in the long time VDT group, the lid margin abnormality score and meiboscore were significantly higher, the expression of meibum was significantly worse compared with the short time VDT group (All *P*<0.0001). These results suggest that long time VDT workers suffer worse meibomian gland function than the short time VDT workers although their aqueous tear production was in normal ranges as measured by Schirmer test in these two groups. The representative images of corneal fluorescein staining and meibomian gland parameters from typical patients of each group were shown in Figure 2. Obviously, the higher scores of corneal fluorescein with worse lid margin abnormality and meibomian grand loss were observed in patients from long time VDT group when compared with short time VDT workers.

**Table 3:** Comparison of Meibomian Gland Parameters in the Long and Short time VDT Groups.

Parameters	Short time VDT (n = 80)	Long time VDT (n = 106)	*P*
Lid margin abnormality score	0.71±0.75	1.46±0.68	<0.0001
Meibum score	0.78±0.67	1.25±0.69	<0.0001
Meiboscore	1.39±0.80	2.32±1.11	<0.0001

Note: Data presented as Mean ± SD of Values.

### Meibomian gland morphology and dysfunction in the long time VDT workers with lower or normal tear volume

**Figure 3:**
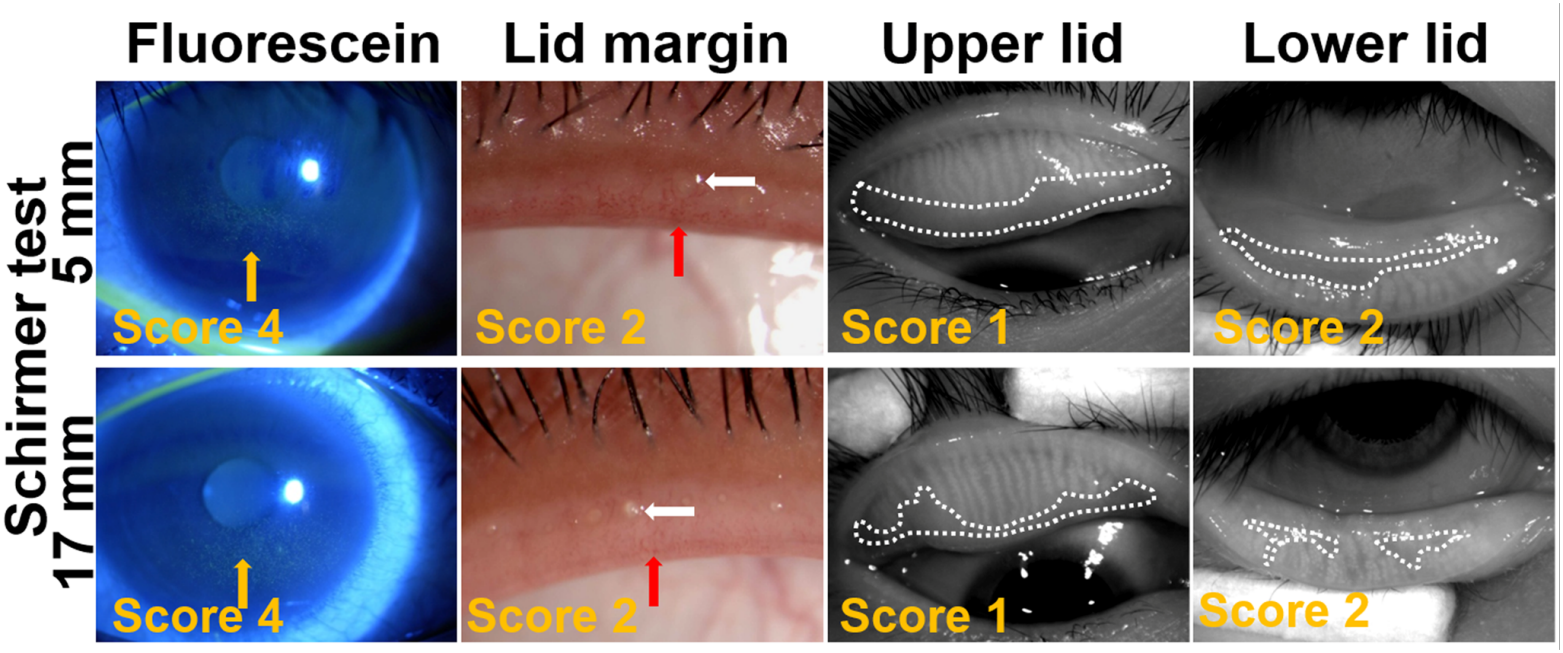
Representative images of fluorescein staining, lid margin and meibography in patients with or without tear volume deficiency in long time VDT group. The score of each image is noted at bottom left. The upper panel shows eyes from low tear volume subgroup: the right eye from a 29-year-old male, VDT work time: 12 h/d; OSDI: 54.55; BUT: 7 s; Schirmer test: 5 mm; the meibum score was 2. The bottom panel shows eyes from normal tear volume subgroup: the right eye from a 37-year-old male, VDT work time: 6 h/d; OSDI: 40.00; BUT: 5 s; Schirmer test: 17 mm; the meibum score was 1. Corneal fluorescein staining is noted by yellow arrows. Vascular engorgement is outlined by red arrow and plugged meibomian gland orifice is outlined by white arrow. The areas of meibomian gland dropout are encircled with dotted white lines.

In order to gain insight into the importance of meibomian gland function in dry eye conditions, the patients from long time VDT group were divided into two subgroups according to Schirmer test: 54 eyes of 27 patients with normal tear volume (Schirmer ≥10 mm) and 52 eyes of 26 patients with low tear volumes (Schirmer <10 mm). As shown in Table 4, the dry eye conditions were similar in these two subgroups as evaluated by OSDI, fluorescein staining and BUT although Schirmer tests were significantly different between them (*P*<0.0001). Interestingly, no statistic difference was found in the lid margin abnormality score, meibum score, and meiboscore between the two subgroups with low or normal aqueous tear volumes. The representative images of corneal fluorescein staining and meibomian gland parameters from typical patients of each subgroup were shown in Figure 3. These findings further demonstrated that the similar dry eye conditions may results from similar meibomian grand functions between these two subgroups although their aqueous teat volumes were dramatically different, low or normal.

**Table 4:** The Comparison of Tear and Meibomian Gland Parameters in the Long Time VDT Workers with Low or Normal Tear Volumes.

Parameters	Schirmer Test (<10 mm, n = 52)	Schirmer Test (>/ = 10 mm, n = 54)	*P*
Schirmer (mm)	4.89±1.98	17.93±6.95	<0.0001
VDT working time (h/d)	8.11±2.37	8.51±2.26	0.407
OSDI	37.92±18.27	36.87±18.34	0.676
BUT (s)	4.73±1.78	5.11±2.89	0.86
Fluorescein score	3.67±2.87	3.78±3.11	0.995
Lid margin abnormality score	1.37±0.63	1.56±0.72	0.114
Meibum score	1.37±0.66	1.15±0.71	0.112
Meiboscore	2.23±1.15	2.41±1.07	0.301

Note: Data presented as Mean ± SD of Values.

### Correlations of meibomian gland dysfunction parameters with dry eye indexes in VDT workers

Spearman correlation analysis showed that VDT working time is significantly correlated with dry eye symptoms OSDI (R = 0.287, *P*<0.0001) and corneal fluorescein score (R = 0.368, *P*<0.0001), inversely correlated with BUT (R = −0.375, *P*<0.0001), but not correlated with Schirmer test values (R = −0.02, *P* = 0.786) in VDT workers. Interestingly, as shown in Table 5, all three MGD parameters were strongly positively correlated with VDT working time (*P*<0.0001) and fluorescein score (*P*<0.0001), inversely correlated with BUT (*P*<0.05), but not correlated with Schirmer test values. Furthermore, OSDI was only correlated with lid margin abnormality score among three MGD parameters in these VDT workers studied (*P*<0.05).

**Table 5:** Simple correlation analysis of 3 Meibomian Gland Dysfunction scores with dry eye parameters in Visual Display Terminal workers.

	Lid margin score		Meibum score		Meiboscore	
	*R*	*P*	*R*	*P*	*R*	*P*
VDT working time (h/day)	0.450	<0.0001	0.318	<0.0001	0.365	<0.0001
OSDI	0.178	0.015	0.052	0.484	−0.088	0.235
Fluorescein score	0.289	<0.0001	0.402	<0.0001	0.373	<0.0001
BUT (s)	−0.299	<0.0001	−0.302	<0.0001	−0.202	0.02
Schirmer (mm)	0.013	0.865	−0.021	0.776	0.004	0.956

Note: *R*, correlation value by Spearman correlation analysis; *P*, significance level in Spearman correlation analysis.

## Discussion

Meibomian gland dysfunction is a chronic and diffuse disorder occurs in meibomian glands, the features are glandular orifices obstruction and/or changes in quality/quantity of the meibomian gland secretion [19]. MGD may affect the tear function; evoke ocular discomfort symptoms and ocular surface diseases, such as dry eye [19]. This investigation was to study the effects of long time VDT working on meibomian glands, and the novel role of MGD in VDT workers suffered with dry eye. Our results revealed that the long time VDT workers suffered more severe ocular discomfort and dry eye conditions than the short time workers although their aqueous tear productions were similar in the normal ranges. However, meibomian glands function was found to be significantly worse in the long time VDT group, suggesting MGD is likely to be responsible for the severity of dry eye syndrome in these subjects.

### VDT workers suffer dry eye disease without significant deficiency of aqueous tear production

Dry eye has been recognized as a primary contributor of ocular disorders in visual display terminal workers [10], [20]. VDT working that force people gazing near and horizontally has been shown to be correlated with reduced blinking frequency and increased ocular surface exposure, both of which accelerate tear evaporation and ocular surface drying [21]. The adverse effects of VDT work were found to be time period dependant; the longer periods of time engaged in VDT working was observed to be correlated with the higher prevalence of dry eye [22], [11], [4]. In the present study of the dry eye patients engaged in VDT work, long time VDT workers were found to suffer more severe dry eye conditions than the short time VDT workers, with more severe ocular discomfort, higher corneal fluorescein staining, and shorter tear film BUT. But the averages of tear volumes measured by Schirmer test were in normal ranges without statistically significant difference between long time and short time VDT groups. Our finding was supported by a previous study of Uchino and associates [11], who reported that average Schirmer value was 18.7±11.7 mm while tear film BUT was much lower to 4.0±2.5 seconds in their study group of young to middle-aged Japanese VDT users. However, it has been lack of further investigation with regard to the mechanism of increased risk for dry eye in long time VDT workers with normal tear volumes. Therefore, we thought that the function of meibomian glands may play a certain role in this controversial situation.

### Meibomian gland function plays a more important role than aqueous tear volume in determining the severity of ocular discomfort and dry eye conditions

Three commonly used parameters of MGD, lid margin abnormality score, meibum expression assessment (meibum score), and the degree of meibomian glands dropout (meiboscore) observed by Keratograph 5 M, were evaluated. Our finding indicated that the long time VDT workers suffered more severe dry eye and also had worse impaired meibomian gland function than the short time group, although their tear volume were similar in normal ranges. The alternations in three MGD parameters were significantly associated with the daily durations of VDT work. Our findings were supported by a previous report that the vast majority of the subjects among VDT workers with the complaint of ocular symptoms exhibited MGD [5].

From the long time VDT workers who suffered dry eye, two subgroups were separated and compared according to their Schirmer tear volumes (<10, or ≥10 mm). We observed that their dry eye conditions, as evaluated by OSDI, corneal fluorescein staining and BUT, were similar between two subgroups regardless the eyes with and without tear volume deficiency. Interestingly, no statistic differences were found in the lid margin abnormality score, meibum score, and meiboscore between the two subgroups. These findings further demonstrated that the function of meibomian glands was likely to be responsible for the severity of ocular discomfort and dry eye conditions in these patients.

The correlation analysis further confirmed that all three MGD parameters were strongly positively correlated with VDT working time and fluorescein score, inversely correlated with BUT, but not correlated with Schirmer test values. These results imply that the alternations in meibomian glands contribute, as least in part, to the tear instability and superficial punctate keratopathy in VDT workers suffered from dry eye. Since the lipids secreted by meibomian glands play a key role in maintaining tear film stability, destruction of meibomian glands may cause tear film instability and subsequently increased tear evaporation [23], [16]. Corneal fluorescein staining was also noted to be associated with incomplete blinking, a kind of blinking pattern which was also important in VDT operation [24]. These findings further support a notion that MGD is a significant cause of ocular surface symptoms and disorders in VDT workers [6].

### Long time VDT work may cause MGD earlier, faster or more severe than tear volume deficiency

The mechanism of MGD in VDT workers is still unclear at present. The delivery of meibum secreted by meibomian glands owing to two kinds of driving force, one is a secretory pressure generated by the continuous secretion of meibum, and another is the muscular action in the tarsus during a blink [25], [26]. Therefore, we propose that the reduced meibum expression associated with decreased blinking rate may contribute to meibum stagnation and gland blockage, subsequent worsening tear instability in VDT users. This hypothesis supports a notion that low blink frequency was associated with a high prevalence of meibomian gland disorder among computer workers who complained ocular discomfort symptoms [27]. On the other hand, radiation was also a potential issue to be concerned although the health issues associated with VDT radiation are not assured. It is known that electromagnetic radiation is able to affect living tissue by destroying chemical bonds and making neutral molecules charged [27]. Among three MGD parameters we studied, the scores of lid margin abnormality was the only variable that was significantly correlated with OSDI, indicating that lid margin abnormality may be an early marker for MGD-associated dry eye.

Using meibography technology, previous studies have discovered that meibomian gland dropout can be affected by various factors, including age, contact lens wear, the use of antiglaucoma eye drops, Sjogren’s syndrome (SS), and graft-versus-host disease (GVHD) [15], [28], [29], [16], [30]. Our findings in this study provided new evidence that longer durations of VDT work may result in similar effects on meibomian gland dropout, but may not cause the deficiency of aqueous tear volume. We further propose a notion that longer durations of VDT work may cause meibomian gland dysfunction earlier, faster or more severe than tear volume deficiency.

However, VDT related ocular surface changes are multifactorial, our study did not cover all confounding factors. In addition to MGD, other investigators have reported that lacrimal gland hypofunction might also contribute to the VDT-associated dry eye in human and rat VDT user models [31].

In conclusion, our findings revealed a unique population in long time VDT workers who suffered ocular discomfort and dry eye syndrome without significant aqueous tear volume deficiency, but with significant alterations in meibomian gland morphology and function. It indicated that longer exposition times to VDT affect meibomian gland structure and function but not tear volume neither Short nor Long term workers, suggesting that MGD is responsible for the severity of dry eye syndrome. As dry eye severity was mainly determined according to OSDI, future studies are important to investigate the comparison and correlation of OSDI degrees (mild, moderate and severe) versus classification of MGD parameters in order to further identify the role of MGD in severity of dry eye. Moreover, longer durations of VDT work may cause MGD earlier, faster, or more severe than tear volume deficiency. Therefore, the examinations of meibomian gland morphology and function, especially meibography, may provide early diagnostic and therapeutic values in dry eye disease.
